# Comparative Evaluation of Twin Block and Forsus Fatigue Resistant Device in the Management of Class II Malocclusion: A Systematic Review

**DOI:** 10.7759/cureus.108162

**Published:** 2026-05-03

**Authors:** Rakesh K Singh, Veera Sawant, Vighanesh Kadam, Wajeeha Mulla, Keval Shroff, Sanpreet S Sachdev

**Affiliations:** 1 Orthodontics and Dentofacial Orthopedics, D.Y. Patil Deemed to be University School of Dentistry, Navi Mumbai, IND; 2 Oral Pathology and Microbiology, Bharati Vidyapeeth (Deemed to be University) Dental College and Hospital, Navi Mumbai, IND

**Keywords:** class ii malocclusion, forsus appliance, functional appliances, mandibular advancement, twin block

## Abstract

Class II malocclusion due to mandibular retrusion is commonly managed with functional appliances, yet the comparative effectiveness of removable and fixed modalities remains uncertain. This systematic review evaluated the skeletal, dentoalveolar, soft-tissue, and airway effects of the Twin Block (TB) appliance and the Forsus Fatigue Resistant Device (FFRD) in the correction of Class II malocclusion. Electronic searching and manual screening identified nine eligible comparative clinical studies for qualitative synthesis. The included studies comprised predominantly nonrandomized designs and together represented 377 participants, with 188 treated using FFRD and 189 using TB. Across the included evidence, both appliances were effective in correcting Class II malocclusion and improving overjet, overbite, and molar relationships. However, the pattern of correction differed between the appliances. TB consistently demonstrated a greater skeletal contribution, with more favorable mandibular advancement, increased mandibular length, and greater improvement in sagittal jaw relationship. In contrast, FFRD treatment relied more on dentoalveolar compensation, particularly proclination of the mandibular incisors. Soft-tissue outcomes, though limited, showed esthetic improvement with both appliances. Airway-related findings were reported in only a few studies and suggested a possible advantage of TB in improving pharyngeal dimensions, although results were not fully consistent across studies. Overall, the available evidence suggests that while both appliances are clinically effective, TB provides a stronger skeletal effect, whereas FFRD achieves correction predominantly through dental adaptation. Further well-designed prospective trials are required to strengthen the comparative evidence base.

## Introduction and background

Class II malocclusion is among the most prevalent orthodontic problems, affecting nearly one-third of patients seeking orthodontic care worldwide [1]. Epidemiological data suggest that its global prevalence in the permanent dentition is approximately 19.5%, with reports indicating that Class II cases may account for 12-49% of orthodontic malocclusions [2,3]. The condition is most commonly attributed to mandibular retrognathism, although other etiological factors such as maxillary prognathism, dentoalveolar discrepancies, and neuromuscular imbalances can also contribute.

Beyond esthetics and occlusion, untreated Class II malocclusion may adversely affect oral function, temporomandibular joint health, and psychosocial well-being, underscoring the need for timely intervention [4]. In addition to skeletal and dental effects, recent evidence synthesis has focused specifically on patient-reported outcomes during functional orthodontic therapy, highlighting the growing importance of discomfort, acceptance, quality of life, and other treatment-experience measures in growing patients [5].

Functional appliances have long been employed to harness growth modification and muscle forces to advance the mandible and correct the sagittal discrepancy in growing patients. Among removable options, the Twin Block (TB) appliance remains one of the most widely prescribed devices [6]. Its design consists of separate upper and lower components with inclined bite blocks that posture the mandible forward, stimulating favorable skeletal and dentoalveolar changes [7]. Clinical studies and surveys have consistently shown its popularity, with more than 75% of orthodontists in the United Kingdom reporting TB as their preferred functional appliance [8].

Despite its effectiveness, success depends heavily on patient compliance, as consistent wear is required to achieve the desired outcomes [9]. Because the clinical effectiveness of removable functional appliances is influenced by wear time, patient acceptance, discomfort, and cooperation, these factors remain important determinants of treatment success [10]. Adjunctive modifications of TB therapy have also been investigated. For example, a randomized controlled trial reported that low-level laser therapy-assisted TB treatment accelerated functional correction and was associated with a greater increase in mandibular length than conventional TB therapy [11].

To overcome compliance-related challenges, fixed functional appliances were developed to apply continuous force independent of patient cooperation [12]. Among these, the Forsus Fatigue Resistant Device (FFRD) has gained popularity as a versatile, chairside-friendly alternative. The FFRD appliance consists of a telescopic push-rod mechanism attached to the mandibular archwire, producing forward mandibular positioning and simultaneous dental correction [13]. Unlike removable devices, it does not interfere significantly with speech and esthetics, making it suitable for adolescents with limited compliance [12,13]. However, concerns remain regarding the extent of true skeletal correction versus dentoalveolar compensation, as well as potential side effects such as proclination of mandibular incisors and soft-tissue irritation.

Given the widespread clinical use of both appliances, a direct comparison of their skeletal, dental, and soft-tissue effects is of paramount importance. Previous individual studies have yielded heterogeneous results, with some highlighting superior skeletal effects with TB, while others reporting comparable or more favorable dentoalveolar changes with FFRD [14,15]. Patient-reported outcomes remain underrepresented in comparative TB versus FFRD studies, despite recent systematic evidence highlighting the importance of pain, discomfort, quality of life, and treatment acceptance during functional therapy [5]. A systematic synthesis of the available evidence is therefore essential to clarify these discrepancies.

In the context of functional appliance therapy, skeletal effects refer to true orthopedic changes in maxillary and mandibular growth, typically assessed using cephalometric parameters such as SNA (Sella-Nasion-A point angle), SNB (Sella-Nasion-B point angle), ANB (A point-Nasion-B point angle), and mandibular length measurements. In contrast, dentoalveolar effects represent tooth movement and positional adaptation, including incisor inclination and molar relationship changes. The primary outcomes of this review were skeletal and dentoalveolar changes following treatment, while secondary outcomes included soft-tissue changes, airway dimensions, and patient-reported outcomes where available. This review and meta-analysis aim to comprehensively evaluate the skeletal and dental outcomes of TB versus FFRD appliances in growing patients with Class II malocclusion, thereby providing evidence-based guidance for clinical decision-making.

## Review

Methodology

The present systematic review and meta-analysis was conducted in accordance with the Preferred Reporting Items for Systematic Reviews and Meta-Analyses (PRISMA) 2020 guidelines [16]. The review protocol adhered closely to the methodological recommendations outlined in the Cochrane Handbook for Systematic Reviews of Interventions (version 5.1.0) and the 4th edition of the Joanna Briggs Institute (JBI) Reviewer’s Manual [17,18]. To ensure transparency and minimize the risk of reporting bias, the protocol was prospectively registered in the PROSPERO database under registration number CRD42024568470. The focused review question was formulated according to the PICOS (Population, Intervention, Comparator, Outcomes, Study design) framework: Is there a difference in the skeletal and dental effects of TB and FFRD appliances in children and adolescents with Class II malocclusion?

Eligibility Criteria

Studies were included if they involved children or adolescents diagnosed with Class II malocclusion requiring functional appliance treatment, irrespective of gender, socioeconomic background, or subdivision of the malocclusion. Eligible interventions included the use of the FFRD, while the comparator was treatment using the TB appliance. The primary outcomes assessed were changes in maxillomandibular skeletal and dental landmarks as evaluated through radiographic methods. Secondary outcomes included dentoalveolar changes, alterations in pharyngeal airway dimensions, and positional modifications of the hyoid bone. A wide range of study designs was considered, including randomized controlled trials, nonrandomized controlled trials, cohort studies, quasi-experimental designs, and cross-sectional studies, provided that full-text articles were accessible or procured within two weeks of contacting the corresponding author. Studies were excluded if they lacked a control group, were case series, review papers, or animal studies, or if only abstracts were available without full-text data.

Search Strategy

A comprehensive and systematic search was performed across major electronic databases, including PubMed, MEDLINE (through OVID), Cochrane Central Register of Controlled Trials (CENTRAL), Scopus, and DOAJ. The search was performed to identify articles from inception until 31 December 2025. Both controlled vocabulary terms (MeSH) and free-text keywords were employed in combination with Boolean operators to maximize sensitivity and specificity. Search terms included variations of “children,” “adolescents,” “Class II malocclusion,” “functional appliance,” “Twin Block,” and “Forsus” or “FFRD.” No restrictions were placed on language, and studies in languages other than English were included if translation was possible. The final search yielded results that were imported into an Elicit systematic review tool for screening and removal of duplicates. The exact search string across all databases is in Appendix A.

Study Selection

The selection of studies was performed in two stages. Initially, the titles and abstracts were independently screened by two reviewers to eliminate irrelevant articles. Full-text versions of potentially eligible studies were then retrieved and evaluated against the predefined inclusion and exclusion criteria. Any disagreements during the selection process were resolved by discussion with a third reviewer. Duplicate publications or multiple reports of the same study were carefully identified and consolidated. Where clarification of study eligibility was required, attempts were made to contact the original authors.

Data Extraction

Data extraction was carried out independently by two reviewers using a standardized and piloted extraction form. The data captured included bibliographic details (author, year, country), study design, sample size, demographic characteristics of participants, details of the interventions and comparators, outcome measures, methods of assessment, results, and conclusions. Additional information on funding sources and potential conflicts of interest was also noted. Extracted data were tabulated and cross-checked to ensure accuracy and consistency. Any disagreements between reviewers were discussed until a consensus was reached.

The primary outcomes for quantitative synthesis were post-treatment skeletal and dentoalveolar cephalometric parameters that were reported in at least two comparative studies with extractable numerical data. Skeletal outcomes included SNA, SNB, ANB, Co-A (Condylion-point A distance), and Co-Gn (Condylion-Gnathion distance). Dentoalveolar outcomes included overjet, overbite, and incisor mandibular plane angle (IMPA). Secondary outcomes included soft-tissue variables, airway dimensions, and other patient-centered outcomes; these were synthesized qualitatively when meta-analysis was not feasible. For pooled analyses, pre- to post-treatment changes were used where comparable data were available.

Risk of Bias Assessment

The methodological quality and risk of bias of the included studies were assessed using validated tools. Randomized controlled trials were appraised with the Cochrane Risk of Bias 2 (RoB 2) tool, which examines domains such as randomization, deviations from intended interventions, missing outcome data, measurement of outcomes, and selective reporting [19]. Nonrandomized studies were evaluated using the ROBINS-I tool, which addresses potential biases due to confounding, participant selection, classification of interventions, deviations from intended interventions, missing data, outcome measurement, and reporting [20]. Assessments were performed independently by two reviewers, with discrepancies resolved through discussion. The certainty of evidence for the pooled outcomes was assessed using the GRADE (grading of recommendations assessment, development, and evaluation) approach [21].

Data Synthesis

Where sufficient homogeneity of outcome reporting was present, quantitative synthesis was performed through meta-analysis. Continuous data were analyzed using mean differences or standardized mean differences (SMDs) with 95% confidence intervals. SMD was selected for the pooled analyses presented in this review because comparable cephalometric outcomes were reported across studies using different scales, baseline conventions, or reporting formats, making standardization preferable to direct mean difference pooling. Statistical heterogeneity was assessed using the chi-square test and quantified with the I² statistic. An I² value of 0-30% was considered low heterogeneity, 30-60% moderate, 50-90% substantial, and above 75% considerable, in line with Cochrane guidelines. A fixed-effects model was employed when heterogeneity was low, while a random-effects model was applied in cases of substantial heterogeneity. Publication bias was assessed through funnel plots where more than 10 studies contributed to an outcome. Subgroup analysis and meta-regression were not performed because of the limited number of studies available for each pooled outcome and the heterogeneity of study design, growth stage assessment, and treatment duration. Formal sensitivity analyses were not undertaken because only a small number of studies contributed to each pooled estimate; this should be considered when interpreting outcomes with high heterogeneity.

Results

**Figure 1:**
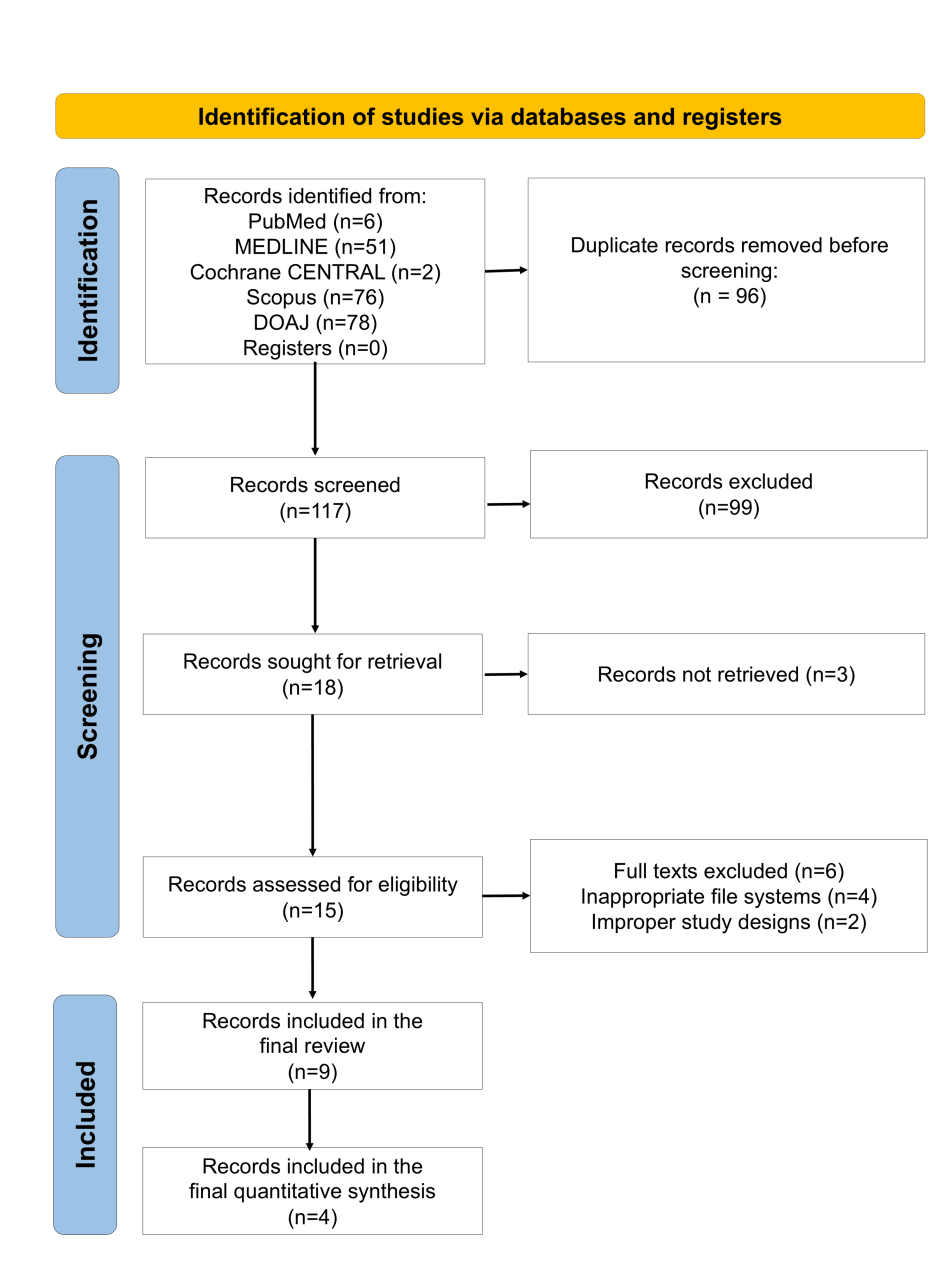
PRISMA 2020 flow diagram indicating the study selection process in the present systematic review PRISMA, Preferred Reporting Items for Systematic Reviews and Meta-Analyses

The electronic database search yielded a pool of potentially relevant records. After removal of duplicates, screening of titles and abstracts, and full-text assessment of potentially eligible articles, nine studies met the inclusion criteria and were included in the qualitative synthesis [22-30]. The study selection process is presented in the PRISMA 2020 flow diagram (Figure 1).

Study Characteristics

The characteristics of the included studies are summarized in Table 1. The evidence base consisted predominantly of comparative nonrandomized clinical studies, with two prospective studies and seven retrospective studies [22-30]. Geographically, the included studies were conducted across multiple settings, with the majority from India [22,23,25,30], and the remainder from Italy, Turkey, Egypt, and Saudi Arabia [24,26-29]. Overall, 377 participants with Class II malocclusion were represented across the included studies, with 188 patients treated using the FFRD appliance and 189 treated using the TB appliance [22-30]. Gender distribution was inconsistently reported, with four studies not specifying sex distribution [22,23,25,26]. Among the studies that did report it, a total of 108 males and 146 females were included [24,27-30]. The outcome domains most frequently assessed were cephalometric skeletal and dentoalveolar changes [22-24,26,27,30], while fewer studies evaluated soft-tissue profile modifications [25] or pharyngeal airway dimensions [28,29]. Across the included literature, a broad pattern was evident in which the TB appliance tended to demonstrate a greater skeletal contribution to Class II correction, whereas the FFRD appliance more often showed correction through dentoalveolar adaptation [22-30].

**Table 1:** Characteristics and principal findings of the included studies FRD: Forsus Fatigue Resistant Device, FFRD: Forsus Fatigue Resistant Device, TB: Twin Block, OJ: overjet, OB: overbite, IMPA: incisor mandibular plane angle, SNA: sella-nasion-A point angle, SNB: sella-nasion-B point angle, ANB: A-N-B angle, Co-A: condylion-point A distance, Co-Gn: condylion-gnathion distance, Wits: Wits appraisal, LAFH: lower anterior facial height, CVMI: cervical vertebral maturation index, CVM: cervical vertebral maturation, IAS: inferior airway space, CBCT: cone beam computed tomography, T0-T1: pretreatment to post-treatment interval, mo: months, y: years

Study	Country	Design	Sample (FRD/comparator)	Age/growth stage	Follow-up	Comparator	Key skeletal findings	Key dental findings	Airway/soft tissue/other findings	Overall interpretation
Mahamad et al. 2012 [22]	India	Retrospective comparative	25/25 (+25 control)	FRD: 11-14 y; TB: 9-12 y	15 mo (FRD), 12 mo (TB)	TB	TB showed greater mandibular length gain; both improved SNB and reduced ANB/Wits; no significant maxillary restraint	Both reduced OJ/OB and achieved Class I; FRD showed greater lower incisor proclination	Both improved soft-tissue profile	TB more skeletal; FRD more dentoalveolar
Tarvade et al. 2014 [23]	India	Comparative clinical study	10 Oct	13-17 y	T0-T1	TB	TB showed greater ANB reduction and mandibular lengthening; both increased SNB; vertical increase was greater with FRD	FRD caused marked IMPA increase; TB showed better incisor control; both improved OJ/OB	Both improved esthetics	TB more effective for mandibular retrognathism; FRD showed more vertical and dentoalveolar effects
Hanoun et al. 2014 [24]	USA-based/Saudi-linked authorship	Retrospective comparative	30/37 (+25 control)	Growing Class II patients; pubertal stage	0.7 y (FRD), 1.3 y (TB)	TB	TB induced greater mandibular advancement and mandibular length increase; FRD changes were less skeletal	FRD showed greater lower incisor proclination; TB showed greater upper incisor retroclination; both corrected molar relation and OJ	Favorable reduction in facial convexity in both	TB more skeletal; FRD mainly dentoalveolar
Chaudhary et al. 2015 [25]	India	Retrospective cephalometric study	10 Oct	TB: 12.5±1.5 y; FRD: 13.5±1.0 y; CVMI 3-4	1 y post-treatment	TB	TB showed greater increase in LAFH	FRD effects were more dentoalveolar	Both improved soft-tissue profile; TB increased LAFH more, while FRD showed greater mentolabial sulcus improvement	Both improved esthetics; TB showed greater skeletal-linked soft-tissue change
Giuntini et al. 2015 [26]	Italy	Retrospective controlled clinical study	36/28 (+27 control)	Mean 12.3-12.4 y; mostly circumpubertal	2.3 y	TB	FRD showed significant maxillary restraint; TB showed greater mandibular advancement and Co-Gn increase	FRD caused significantly greater mandibular incisor proclination	Similar treatment success in both groups	TB more skeletal; FRD more dentoalveolar with maxillary restraint
Gulec and Goymen 2018 [27]	Turkey	Retrospective comparative	15/15 (+10 control)	Mean 12-13 y; CVMI III-V	0.5 y (FRD), 1.1 y (TB)	TB	Both increased Co-Gn; TB also showed maxillary restriction and posterior mandibular rotation; FRD correction was mainly via mandibular growth	FRD produced greater mandibular incisor proclination; TB showed greater overjet reduction through combined upper incisor retroclination and lower incisor proclination	No significant soft-tissue difference	TB more skeletal with maxillary effect; FRD more dentoalveolar
Alhammadi et al. 2019 [28]	Egypt	Prospective controlled clinical trial	21/31 (+22 control)	Growing skeletal Class II patients	Active phase+3-mo retention (TB); FRD until edge-to-edge incisal correction	TB	TB showed more evident skeletal changes than FFRD	FRD was associated more with dentoalveolar correction	3D CBCT study; TB showed more favorable pharyngeal airway changes than FFRD	TB superior for skeletal and airway improvement
Yavan et al. 2021 [29]	Turkey	Retrospective comparative	25/25	TB: 12.5±3.4 y; FFRD: 13.5±2.8 y; CVMI 3-4	11 mo (TB), 8 mo (FFRD)	TB	TB showed significantly greater mandibular advancement; FFRD showed significant maxillary inhibition	Both proclined lower incisors and reduced OJ/OB	TB showed significant increase in IAS, oropharyngeal area, and forward/downward hyoid movement; no significant between-group difference in overall uvulo-glossopharyngeal dimensions	TB more favorable for mandibular advancement and airway changes
Pawar et al. 2023 [30]	India	Prospective comparative study	14/14	Mean 11±1.46 y; CVM2-CVM3	1 y	PowerScope	Removable appliances showed greater skeletal effects overall	Fixed appliances mainly produced dentoalveolar correction	Better facial profile improvement with removable appliances overall	Among fixed appliances, FRD and PowerScope were broadly comparable; removable appliances were more skeletal

Skeletal Outcomes

Eight studies assessed skeletal outcomes using cephalometric analysis [22-24,26-30]. Across these studies, the TB appliance generally showed a stronger tendency to enhance mandibular advancement and improve sagittal skeletal relationships [22-24,26,27,30]. This was reflected in more favorable changes in parameters such as SNB, ANB, mandibular length, and Wits appraisal in several studies [22,23,26,27,30]. In contrast, the FFRD appliance was more often associated with a comparatively smaller skeletal effect, with some studies suggesting that its action relied less on true mandibular growth modification and more on dentoalveolar compensation [22,24,26,27,30]. Although the magnitude of skeletal change varied between studies because of differences in study design, age groups, growth stage, and duration of treatment, the overall direction of findings favored the TB appliance as the more skeletally effective modality for the correction of mandibular retrognathism in Class II malocclusion [22-24,26-30].

Dentoalveolar Outcomes

Dentoalveolar effects were reported in the majority of included studies and showed a relatively consistent pattern [22-24,26,27,30]. Both appliances produced meaningful improvement in overjet, overbite, and molar relationships, indicating that both were clinically effective for Class II correction [22-24,26,27,30]. However, the FFRD appliance showed a stronger association with proclination of the mandibular incisors, suggesting a more pronounced dentoalveolar mode of correction rather than a predominantly skeletal one [22-24,26,27]. This finding emerged as one of the most consistent trends across studies [22-24,26,27]. The TB appliance, although also capable of producing dentoalveolar changes, appeared to do so with a greater accompanying skeletal component [22,23,26,27,30]. Thus, while both appliances achieved clinically useful occlusal correction, the mechanism of that correction differed, with FFRD demonstrating a greater reliance on lower incisor proclination and dental compensation [22-24,26,27,30].

Soft-Tissue Changes

Soft-tissue outcomes were more limited in the included literature and were specifically evaluated in one study [25]. Both the TB and FFRD appliances were found to improve facial esthetics and profile balance [25]. The TB appliance demonstrated a greater influence on lower anterior facial height, whereas the FFRD appliance showed a more notable effect on the mentolabial sulcus [25]. Although the available evidence for soft-tissue outcomes was limited, the findings suggested that both appliances contributed positively to facial profile improvement, though with somewhat different patterns of soft-tissue adaptation [25].

Airway Evaluation

Pharyngeal airway changes were assessed in two included studies [28,29]. One study reported that the TB appliance produced more evident skeletal improvement along with more favorable enlargement of the pharyngeal airway compared with FFRD therapy [28]. In contrast, another study found no statistically significant between-group difference in airway dimensions following treatment [29]. Taken together, these findings suggest that airway effects remain less consistent than skeletal and dentoalveolar effects. While the available evidence indicates that TB therapy may have a more favorable influence on airway dimensions in some populations [28], the limited number of studies and heterogeneity in methods and patient characteristics mean that these findings should be interpreted cautiously [28,29].

Overall Synthesis of Findings

Overall, the qualitative synthesis demonstrated that both the TB and FFRD appliances are effective in the treatment of Class II malocclusion [22-30]. However, the pattern of correction differed between the appliances. The TB appliance more frequently showed a skeletal mode of action, particularly through greater mandibular advancement and improvement in sagittal jaw relationships [22-24,26-30], whereas the FFRD appliance more commonly demonstrated dentoalveolar correction, especially through proclination of the mandibular incisors [22-24,26,27]. Soft-tissue improvements were reported with both appliances [25], and airway findings were limited and somewhat variable [28,29]. Despite heterogeneity in study design and outcome reporting, the included studies collectively supported the view that the TB appliance offers a stronger skeletal contribution, while FFRD correction is achieved more through dental adaptation [22-30].

Risk of Bias

**Figure 2:**
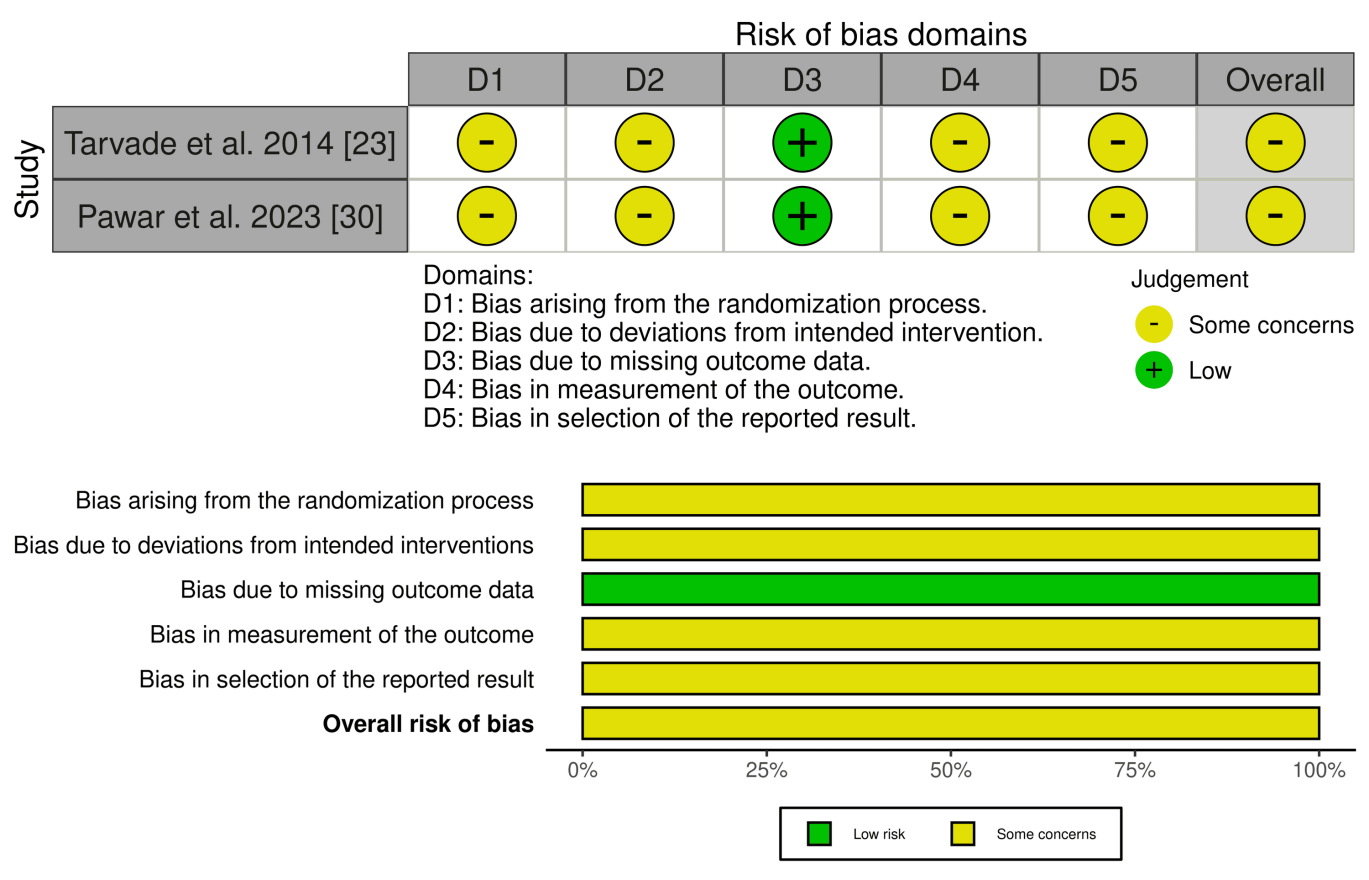
Risk of bias assessment of randomized studies using the Cochrane RoB 2 tool Source: [23,30] RoB 2, Risk of Bias 2

Among the two randomized studies, both were judged as having some concerns overall (Figure 2) [23,30]. The main limitation was insufficient detail regarding sequence generation and allocation concealment, which lowered confidence in the randomization process [23,30]. Missing outcome data were not a major concern in either study, but limited reporting on blinding, adherence to intended interventions, and prespecification of analyses introduced additional concerns in the domains of deviations from intended interventions, measurement of outcomes, and selection of the reported result [23,30].

**Figure 3:**
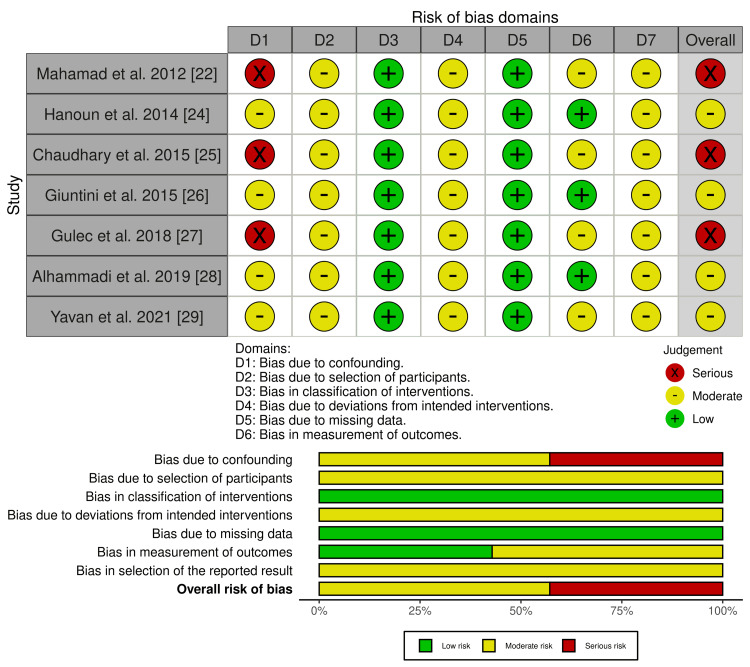
Risk of bias assessment of nonrandomized studies of interventions using the ROBINS-I tool Source: [22,24-29]

Among the seven nonrandomized studies (Figure 3), the overall risk of bias ranged from moderate to serious [22,24-29]. The most important limitation was bias due to confounding, since treatment allocation was not randomized and may have been influenced by factors such as growth stage, appliance indication, baseline malocclusion severity, or clinician preference [22,24-29]. Additional concerns arose from participant selection and the retrospective design of most studies, while bias due to missing data was generally low because most studies reported complete pre- and post-treatment records for the analyzed sample [22,24-29]. Measurement bias was lower in studies using standardized cephalometric or cone beam computed tomography (CBCT) protocols, but remained a concern where assessor blinding or calibration procedures were unclear [22,24-29]. Overall, four studies were judged at moderate risk of bias and three studies at serious risk of bias, indicating that the nonrandomized evidence should be interpreted with appropriate caution [22,24-29].

Meta-analysis

Maxillary Skeletal Parameters

**Figure 4:**
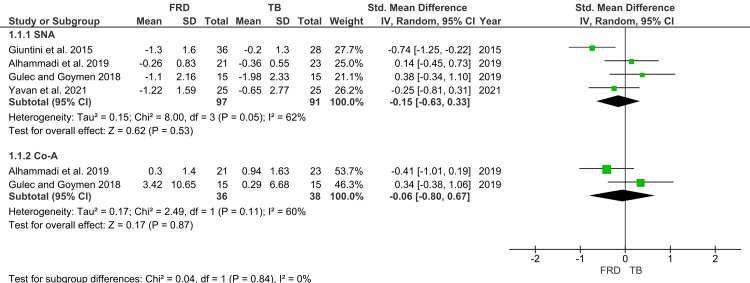
Forest plot for maxillary parameters Source: [26-29] FRD, Forsus Fatigue Resistant Device; TB, Twin Block; SNA, Sella-Nasion-A point angle; Co-Gn, Condylion-point A distance

Four studies contributed to the pooled analysis of post-treatment SNA values, representing 97 participants in the FFRD group and 91 in the TB group (Figure 4) [26-29]. The pooled SMD suggested a smaller reduction in SNA with FFRD compared to TB (SMD=-0.15; 95% CI: -0.63 to 0.33), although the difference was not statistically significant (p>0.05). Heterogeneity across these studies was moderate to high, and thus a random-effects model was applied. For Co-A values, two studies provided data on 36 FFRD and 38 TB patients. The pooled estimate (SMD=-0.06; 95% CI: -0.80 to 0.67) again showed no statistically significant differences between the appliances (p>0.05).

Mandibular Skeletal Parameters

**Figure 5:**
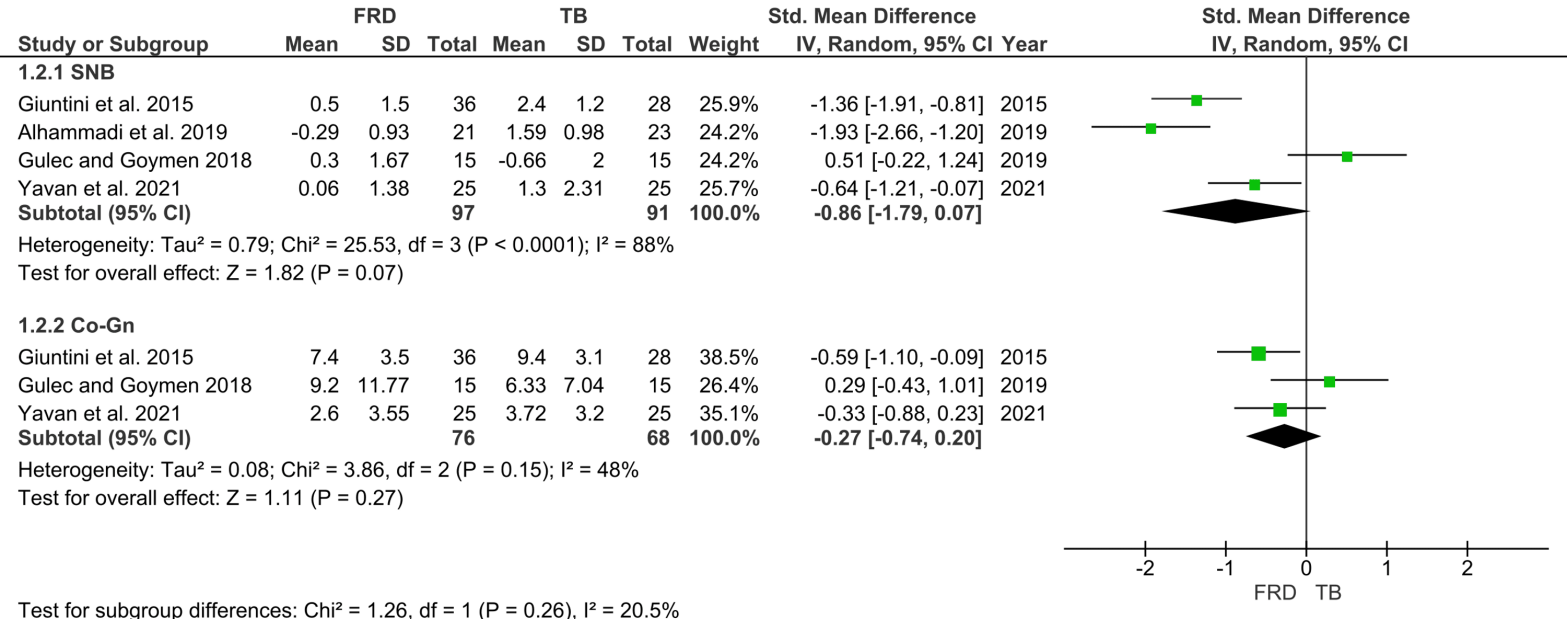
Forest plot of maxillary parameters Source: [26-29] FRD, Forsus Fatigue Resistant Device; TB, Twin Block; SNB, Sella-Nasion-B point angle; Co-Gn, Condylion-Gnathion distance

Four studies reported post-treatment SNB values, with 97 participants in the FFRD group and 91 in the TB group (Figure 5) [26-29]. The pooled effect size favored TB, indicating a greater increase in SNB compared to FFRD (SMD=-0.86; 95% CI: -1.79 to 0.07). While this trend suggested superior mandibular advancement with TB, the result did not achieve statistical significance (p>0.05). Substantial heterogeneity was detected (I²=88%). For Co-Gn, three studies including 144 participants were analyzed. The pooled SMD was -0.27 (95% CI: -0.74 to 0.20), again suggesting greater mandibular elongation with TB, but without statistical significance.

Skeletal Relationship

**Figure 6:**
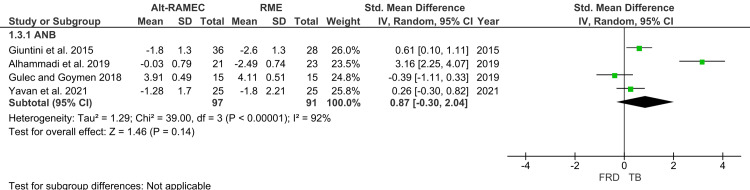
Forest plot of skeletal landmark - ANB angle Source: [26-29] FRD, Forsus Fatigue Resistant Device; TB, Twin Block; ANB, A point-Nasion-B point angle

Pooled analysis of four studies reporting ANB revealed an SMD of 0.87 (95% CI: -0.30 to 2.04), suggesting a tendency for FFRD to produce greater reductions in ANB compared to TB (Figure 6) [26-29]. However, the wide confidence interval and lack of statistical significance (p>0.05) indicate considerable variability across studies (I²=92%). These results highlight inconsistent effects of the two appliances on sagittal skeletal relationships.

Dentoalveolar Outcomes

**Figure 7:**
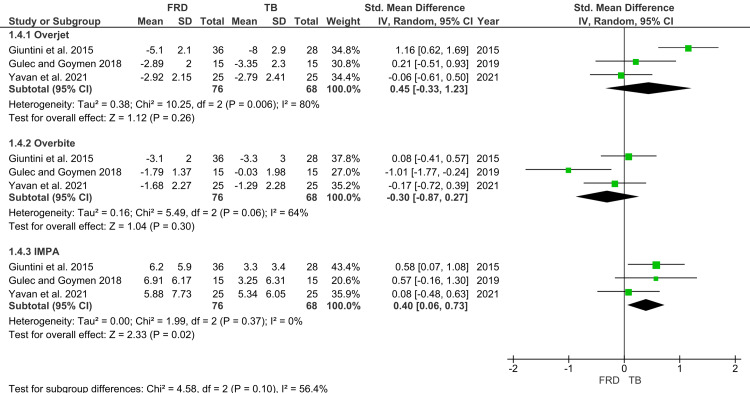
Forest plot of dentoalveolar landmarks Source: [26,27,29] FRD, Forsus Fatigue Resistant Device; TB, Twin Block; IMPA, incisor mandibular plane angle

Overjet changes were assessed in three studies, encompassing 144 patients. FFRD treatment was associated with a greater reduction in overjet compared to TB (SMD=0.45; 95% CI: -0.33 to 1.23), though this finding did not reach statistical significance (p>0.05). Heterogeneity remained high (I²=80%). Similarly, pooled analysis of overbite changes across three studies demonstrated no significant differences between appliances (SMD=-0.30; 95% CI: -0.87 to 0.27). In contrast, analysis of IMPA changes across three studies revealed a statistically significant effect. FFRD produced greater proclination of mandibular incisors than TB (SMD=0.40; 95% CI: 0.06 to 0.73; p<0.05). This was the only parameter where a statistically significant difference was consistently observed between the two appliances. The forest plots for the three parameters are depicted in Figure 7 [26,27,29].

Certainty of Evidence

Overall, the certainty of evidence was judged to be very low for all key outcomes (Table 2). This was primarily because the quantitative synthesis was based predominantly on nonrandomized comparative studies, which were subject to confounding and selection bias, and because several pooled outcomes showed substantial inconsistency and imprecision. Maxillary outcomes such as SNA and Co-A were rated as very low certainty because of serious risk of bias, small sample sizes, and wide confidence intervals. Mandibular and intermaxillary skeletal outcomes, including SNB, Co-Gn, and ANB, were also rated as very low certainty, with additional downgrading for marked heterogeneity, particularly for SNB and ANB. Dentoalveolar outcomes, including overjet and overbite, likewise showed very low certainty because of serious inconsistency and imprecision. Although IMPA was the only pooled parameter to show a statistically significant difference favoring greater mandibular incisor proclination with FFRD, the certainty of evidence for this finding was still judged to be very low due to the observational nature of the contributing studies and limited sample size. Overall, the GRADE assessment indicates that the pooled estimates should be interpreted cautiously and that further well-designed randomized clinical trials are likely to have an important impact on the certainty of the evidence and may change the estimated effects.

**Table 2:** GRADE certainty of evidence for key pooled outcomes GRADE, grading of recommendations assessment, development, and evaluation; SNA, Sella-Nasion-A point angle; Co-Gn, Condylion-point A distance; SNB, Sella-Nasion-B point angle; Co-Gn, Condylion-Gnathion distance; ANB, A point-Nasion-B point angle; IMPA, incisor mandibular plane angle

Outcome	No. of studies	Study design	Risk of bias	Inconsistency	Indirectness	Imprecision	Publication bias	Overall certainty	Interpretation
SNA	4	Observational comparative studies	Serious	Serious	Not serious	Serious	Undetected	Very low	Evidence is very uncertain regarding differences between TB and FFRD for maxillary position.
Co-A	2	Observational comparative studies	Serious	Serious	Not serious	Serious	Undetected	Very low	Evidence is very uncertain regarding differences in maxillary length between appliances.
SNB	4	Observational comparative studies	Serious	Very serious	Not serious	Serious	Undetected	Very low	Evidence is very uncertain regarding the apparent greater mandibular advancement with TB.
Co-Gn	3	Observational comparative studies	Serious	Serious	Not serious	Serious	Undetected	Very low	Evidence is very uncertain regarding differences in mandibular lengthening between appliances.
ANB	4	Observational comparative studies	Serious	Very serious	Not serious	Serious	Undetected	Very low	Evidence is very uncertain regarding intermaxillary sagittal correction.
Overjet	3	Observational comparative studies	Serious	Very serious	Not serious	Serious	Undetected	Very low	Evidence is very uncertain regarding differences in overjet reduction between appliances.
Overbite	3	Observational comparative studies	Serious	Serious	Not serious	Serious	Undetected	Very low	Evidence is very uncertain regarding differences in overbite reduction between appliances.
IMPA	3	Observational comparative studies	Serious	Not serious	Not serious	Serious	Undetected	Very low	Evidence is very uncertain, although FFRD appears to be associated with greater mandibular incisor proclination.

Discussion

This systematic review and meta-analysis found that both TB and FFRD appliances were effective in correcting Class II malocclusion, but their pattern of correction differed. TB showed a greater skeletal contribution, particularly in mandibular advancement and sagittal jaw correction, whereas FFRD relied more on dentoalveolar adaptation, especially mandibular incisor proclination. Soft-tissue and airway findings were comparatively limited and less consistent across studies. Class II malocclusion associated with mandibular retrusion remains one of the most common sagittal discrepancies managed in orthodontic practice, and both removable and fixed functional appliances continue to play an important role in its correction [31].

Appliance selection is influenced by multiple factors, including skeletal pattern, growth status, expected patient compliance, treatment duration, and the desired balance between skeletal and dentoalveolar correction. In the present systematic review, the treatment effects of the TB appliance and the FFRD were evaluated across different populations and study designs. The findings indicate that both appliances were effective in correcting Class II malocclusion. However, their pattern of action differed, with TB demonstrating a greater skeletal contribution and FFRD showing a stronger dentoalveolar component [22-30]. Variations in treatment protocols, including adjunctive therapies such as low-level laser application, may also influence treatment outcomes in TB therapy [12].

The pooled qualitative evidence suggests that maxillary skeletal changes following treatment with either appliance were generally limited, indicating that sagittal correction was achieved primarily through mandibular advancement, dentoalveolar adaptation, or a combination of both [22-30]. This is in agreement with previous literature showing that functional correction in Class II patients is often more dependent on mandibular and dentoalveolar responses than on substantial maxillary restriction [32]. Across the included studies, TB more consistently demonstrated greater increases in SNB, mandibular length, and overall sagittal jaw correction, supporting its role as a more effective growth-modifying appliance in appropriately timed cases [22-24,26-30,33]. A recent randomized clinical trial comparing mini-implant-supported TB with conventional TB also reported greater skeletal correction and reduced dentoalveolar side effects with skeletal anchorage, further supporting the skeletal potential of TB-based therapy [34].

Recent randomized evidence has further strengthened the skeletal rationale for TB therapy, as mini-implant-supported TB was reported to produce greater skeletal correction with reduced dentoalveolar side effects compared with conventional TB therapy [34]. In contrast, FFRD correction tended to rely more heavily on dentoalveolar adaptation, particularly proclination of the mandibular incisors, which emerged as the most consistent side effect associated with this appliance [22-24,26,27]. One proposed approach to minimize this unwanted lower incisor proclination is the use of skeletal anchorage with TB therapy, as recent randomized evidence on mini-implant-supported TB demonstrated reduced dentoalveolar side effects compared with the conventional appliance [34]. This pattern is consistent with previous reports describing fixed functional appliances as effective compliance-free modalities, but with greater dental compensation and less pronounced skeletal enhancement than removable orthopedic appliances [35,36].

With regard to intermaxillary correction, TB generally produced greater improvement in sagittal skeletal relationships, particularly in reductions of the ANB angle and improvement in mandibular position relative to the cranial base [22-30]. Although both appliances achieved clinically meaningful reductions in overjet and overbite, the mechanisms differed. In the TB group, correction appeared to be achieved through a more balanced combination of skeletal and dentoalveolar effects, whereas in the FFRD group, dental movements, especially lower incisor proclination, played a larger role [22-30]. These differences are clinically relevant, as TB may be more suitable in patients in whom skeletal correction is the primary objective, while FFRD may be advantageous when compliance is a concern and a dentoalveolar mode of correction is acceptable.

Soft-tissue outcomes were reported less frequently, but the available evidence suggests that both appliances improved facial esthetics [25]. This limited evidence within the present review should be interpreted alongside recent systematic evidence showing that external soft-tissue responses have increasingly been evaluated across removable and fixed functional appliances in growing Class II patients [5]. TB appeared to exert a greater influence on lower anterior facial height, whereas FFRD showed a more marked effect on the mentolabial sulcus [25].

Similarly, airway-related findings were limited and somewhat heterogeneous. Some studies suggested that TB may have a more favorable effect on pharyngeal airway dimensions, likely secondary to greater mandibular advancement and associated soft-tissue adaptation, whereas FFRD showed either smaller changes or no significant between-group differences [28,29]. These findings are consistent with broader evidence suggesting that orthopedic and functional interventions can influence upper airway dimensions, particularly in growing patients undergoing maxillary or mandibular advancement [37]. Patient-reported outcomes remain underrepresented in comparative TB versus FFRD studies, despite recent systematic evidence highlighting the importance of pain, discomfort, quality of life, and treatment acceptance during functional therapy [5]. These findings should, thus, be interpreted cautiously because airway outcomes were reported in only a small subset of studies and with variable assessment methods. Attempts to reduce early treatment-related discomfort have also been investigated. However, one randomized clinical trial found that local application of glucosamine sulfate and chondroitin sulfate did not significantly alter temporomandibular joint spaces, pain, or tension levels during removable functional treatment [38].

This review has several limitations that must be acknowledged. The included studies were heterogeneous in design, sample size, age range, growth stage, treatment duration, and outcome assessment, which limits the strength of direct comparisons [22-30]. Most studies were nonrandomized, increasing susceptibility to selection bias and confounding. In addition, several studies had relatively small samples, and outcomes such as airway changes and soft-tissue effects were underrepresented [25,28,29]. Another limitation is that much of the evidence relied on two-dimensional cephalometric measurements, which may not fully capture complex skeletal, airway, and soft-tissue changes. Furthermore, patient compliance, which is particularly relevant for TB therapy, was seldom quantified, despite its likely influence on treatment response.

Within these limitations, the available evidence suggests that TB offers a greater skeletal contribution to Class II correction, whereas FFRD produces correction predominantly through dentoalveolar adaptation. Future well-designed prospective and randomized studies with standardized protocols and outcome reporting are needed to provide more definitive comparative evidence for functional appliance therapy in Class II malocclusion.

## Conclusions

TB and FFRD appliances are both effective in the management of Class II malocclusion, though they achieve correction through different mechanisms. The TB exerts its primary influence by stimulating mandibular growth and producing more favorable skeletal modifications, whereas the FFRD relies predominantly on dentoalveolar compensation, with a characteristic proclination of the mandibular incisors. While improvements in overjet, overbite, and facial esthetics were observed with both appliances, the TB demonstrated a consistent advantage in skeletal advancement and airway changes, whereas FFRD offered the benefit of reduced dependence on patient compliance. Given the heterogeneity and predominance of nonrandomized studies in the current evidence base, future high-quality randomized controlled trials with standardized reporting are essential to validate these findings and provide stronger guidance for clinical decision-making.

## Appendices

Appendix A

**Table 3:** Search strings in different databases

Database	Search	Number of articles obtained
PubMed	(((children OR child OR adolescents) AND (class ii malocclusion*)) AND (forsus appliance OR Forsus Fatigue Resistance Device OR FFRD appliance)) AND (twin block appliance)	6
PMC/MEDLINE	((((("child"[MeSH Terms] OR "child"[All Fields]) OR ("child"[MeSH Terms] OR "child"[All Fields] OR "children"[All Fields])) OR ("adolescent"[MeSH Terms] OR "adolescent"[All Fields] OR "adolescents"[All Fields])) AND "malocclusion, angle class ii"[MeSH Terms]) AND ((functional[All Fields] AND appliance[All Fields]) AND (("twins"[MeSH Terms] OR "twins"[All Fields] OR "twin"[All Fields]) AND block[All Fields] AND appliance[All Fields]))) AND (((forsus[All Fields] AND	51
Scopus	("fatigue"[MeSH Terms] OR "fatigue"[All Fields]) AND resistance[All Fields] AND ("equipment and supplies"[MeSH Terms] OR ("equipment"[All Fields] AND "supplies"[All Fields]) OR "equipment and supplies"[All Fields] OR "device"[All Fields])) OR (FFRD[All Fields] AND appliance[All Fields])) OR (forsus[All Fields] AND appliance[All Fields]))	76
Cochrane CENTRAL	children AND class II malocclusion AND twin block appliance AND forsus appliance	2
DOAJ	Child OR children AND class II malocclusion AND functional appliance AND forsus appliance OR FFRD AND twin block appliance	78
